# Impact of a Novel Radiation Protection System on Door-to-Balloon Time During STEMI Intervention

**DOI:** 10.1016/j.jscai.2025.104108

**Published:** 2025-12-18

**Authors:** Osama Hallak, Mitchell Mehringer, Hamza Alkowatli, Jonathan Wayne Lowery, James B. Hermiller

**Affiliations:** a Department of Interventional Cardiovascular Medicine, Ascension St. Vincent Heart Center, Indianapolis, Indiana; b Department of Internal Medicine, Ascension St. Vincent Hospital, Indianapolis, Indiana; c Department of Internal Medicine, HCA Blake Hospital, Bradenton, Florida; d Department of Physiology and Pharmacology, Wood College of Osteopathic Medicine, Marian University, Indianapolis, Indiana; e Division of Academic Affairs, Marian University, Indianapolis, Indiana

**Keywords:** catheterization lab safety, door-to-balloon time, enhanced radiation protection system, percutaneous coronary intervention, Rampart system, ST-elevation myocardial infarction

## Abstract

**Background:**

Prolonged radiation exposure remains a critical occupational hazard in the catheterization lab, typically mitigated by lead aprons, which contribute to orthopedic injury. Recent innovations like the Rampart M1128 enhanced radiation protection system (Rampart IC) aim to reduce these risks, but their impact on door-to-balloon (D2B) time in ST-elevation myocardial infarction (STEMI) has not been evaluated.

**Methods:**

We conducted a single-center retrospective cohort study comparing D2B times among 174 STEMI patients undergoing percutaneous coronary intervention: 87 treated using lead aprons and 87 treated utilizing the Rampart M1128 system. Patients were randomly selected from a pool of 298. Analyses were adjusted for potential confounders, including bleeding, use of vasopressors, intubation, mechanical support, electrical instability, access type, and prior revascularization.

**Results:**

Unadjusted median D2B time was 31.0 minutes in the Rampart group vs 31.1 minutes in the lead apron group. Adjusted analysis yielded a mean D2B difference of 1.51 minutes (95% CI, –0.86 to 3.87; *P* = .21), well within the prespecified 5-minute noninferiority margin. No statistically significant differences were observed across expanded models or one-at-a-time covariate models. The presence of shock or respiratory failure did not prolong D2B times in the Rampart group.

**Conclusions:**

Implementation of an enhanced radiation protection system was noninferior to standard lead apron use regarding D2B time in STEMI patients. This suggests such systems can enhance operator safety without compromising the quality of patient care. Broader adoption may be justified to reduce occupational harm while maintaining high-quality cardiovascular outcomes.

## Introduction

Door-to-balloon (D2B) time is a metric used by interventional cardiologists for primary percutaneous intervention in patients with ST-elevation myocardial infarction (STEMI).^1^ Current 2025 American College of Cardiology/American Heart Association guidelines recommend timely reperfusion with a D2B time of 90 minutes or less, whereas the 2017 European Society of Cardiology guidelines recommend a D2B time of 60 minutes or less. Certain data suggest that shortening D2B time by <45 minutes was significantly associated with reduced mortality and vice versa.^2^, ^3^, ^4^, ^5^ Researchers are continuously studying a variety of factors that influence D2B time, with the goal of identifying key improvements that can help expedite the process and ultimately enhance the quality of patient care by improving outcomes for individuals experiencing critical cardiac events.^6^, ^7^, ^8^

Radiation exposure remains a significant occupational hazard for medical professionals involved in fluoroscopically guided cardiovascular procedures. Prolonged exposure raises serious health concerns, increasing the risk of conditions such as cataracts and cancer among interventional staff.^9^ Currently, lead aprons are the standard method of reducing radiation exposure, however, at the cost of continuous musculoskeletal strain.^10^ Notably, a 2023 survey of interventional cardiologists reported a high prevalence of orthopedic injuries (59.8%), underscoring the substantial occupational hazards faced by operators in the catheterization laboratory. Many interventional cardiologists worldwide have experienced reduced procedural capacity or taken leave due to occupational injuries, including joint-related issues such as hip, knee, ankle, wrist, elbow, and shoulder injuries. Spinal problems were also common, with reports of both cervical and lumbar spine injuries requiring surgical intervention. A significant number indicated that better prevention and management of these hazards would encourage them to remain in practice longer, with some suggesting that they could extend their careers by a decade or more.^10^ As physicians, our foremost priority is ensuring patient safety and providing the highest quality of care. For previous generations, this commitment often came at the expense of their own health. However, recent advancements to reduce the hazards of working in the cardiac catheterization laboratory, namely enhanced radiation protection systems (ERPS), have demonstrated a significant reduction in physician radiation exposure and orthopedic strain.^11^, ^12^, ^13^ Although these innovations offer crucial benefits to health care providers, it is essential to assess their impact on the quality of care delivered to STEMI patients during their most critical moments. This study aimed to evaluate the effect of implementing an ERPS on D2B time in patients with STEMI.

## Methods

A total of 385 patients with STEMI treated in the cardiac catheterization lab were analyzed. D2B times of each patient were recorded in HH:MM:SS format and converted into total minutes for analysis.

Data from 298 patients suffering from STEMI who were treated in our facility with percutaneous intervention by catheterization lab personnel using lead aprons for radiation protection were available. These data from STEMI cases with staff utilizing lead aprons were collected over a 3-year period. Of these patients, 87 were randomly selected for evaluation via simple random sampling using the RAND function in Excel (Microsoft Corp), which assigned a random numerical value between 0 and 1 to each patient. Patients were then ordered in ascending order based on this random numerical value, and the first 87 were selected for the analysis. No stratification or matching was applied. Baseline characteristics were compared between the groups using χ^2^ tests (except for age, which was tested using the Mann-Whitney *U* test). Baseline characteristics are reported as median (IQR).

Data collected over 11 months from 87 STEMI patients treated with the Rampart M1128 ERPS (Rampart IC) were available. This process did not require random selection. Limited data were available as the system was only recently incorporated. All data from STEMI patients treated with this system during the timeline studied were included in the analysis.

Using 87 patients treated with each approach, and a margin of 5 minutes, provided >90% power to detect noninferiority. Five minutes was selected in order to achieve an appropriate power calculation, given the limited data available due to the recent incorporation of ERPS into STEMI care. We believe that given the associated occupational safety benefits of the radiation protection systems, a delay of <5 minutes would be acceptable and clinically irrelevant, although this is not based on any published data.

A Shapiro-Wilk test was performed, and it was found that data from each group were not normally distributed; therefore, the nonparametric Mann-Whitney *U* test was used for primary comparisons. Unadjusted group comparisons were performed using the Mann-Whitney *U* test, and continuous variables are reported as median (IQR); Hodges-Lehmann shift estimates with 95% CI were also calculated. Baseline categorical variables were compared with χ^2^ or Fisher exact tests, as appropriate. For adjusted analyses, we fit multivariable linear regression models including bleeding, acute kidney injury, intubation, and access site. Full regression estimates for the primary adjusted model are presented in Table 2. As an additional sensitivity analysis, we refit the adjusted model using generalized linear models with gamma and inverse Gaussian distributions (log links). Multivariable regression models were used to assess for differences in patient or procedural characteristics that may have influenced variations in D2B time. An expanded model (full covariate adjustment) was used to adjust for patient demographics, cardiac risk factors, access type, and in-hospital complications (bleeding, vasopressor requirements, intubation, mechanical support, or electrical instability). Univariate covariate analysis (one-at-a-time) was also performed to evaluate the influences of bleeding, intubation, and access (femoral vs radial) individually and help isolate the impact of each factor, ensuring transparency about how each adjustment affected the overall comparison.

To explore user differences resulting in improved D2B times with increased use of an ERPS, that is, a “learning curve,” a simple linear regression using procedure date as the predictor and D2B time as the dependent variable was conducted.

## Results

A total of 298 patients in the lead apron category (mean age, 63.9 years) were available; 87 of those in the lead apron category (mean age, 64.0 years) and 87 in the ERPS category (mean age, 64.7 years) were analyzed (Table 1). A Shapiro-Wilk test confirmed the data were not normally distributed (lead apron *P* = .04, ERPS *P* = .001); therefore, the analysis was performed using the Mann-Whitney *U* test. Median D2B time was 31.0 minutes (IQR, 25.0-37.5) for the ERPS group versus 31.10 minutes (IQR, 26.6-34.3) for the lead apron group (Mann–Whitney U = 3948, *P* = .72). The Hodges-Lehmann shift estimate indicated a group difference of 0.35 minutes (95% CI, –1.82 to 2.88), confirming no significant difference between the groups. In the adjusted model, use of the ERPS was associated with a nonsignificant difference in D2B time (β = 1.36 minutes; 95% CI, −1.00 to 3.72; *P* = .26), and covariate estimates were directionally consistent with prior expectations (Table 2). Re-estimation of the adjusted analysis using generalized linear models (gamma and inverse Gaussian with log links) yielded the same conclusion, with no significant group effect and similar covariate patterns (data not shown).

**Table 1:** Baseline characteristics of STEMI patients treated with lead aprons vs enhanced radiation protection system.

Characteristic	Lead aprons (n = 87)	ERPS (n = 87)	*P* value
Male sex	59 (67.8%)	59 (67.8%)	>.99
Hypertension	51 (58.6%)	55 (63.2%)	.58
Diabetes mellitus	27 (31.0%)	22 (25.3%)	.53
Hyperlipidemia	45 (51.7%)	54 (62.1%)	.19
Ejection fraction <50%	3 (3.4%)	6 (6.9%)	.33
Cerebrovascular accident	2 (2.3%)	8 (9.2%)	.50
Tobacco use	57 (65.5%)	52 (59.8%)	.60
Peripheral artery disease	7 (8.0%)	4 (4.6%)	.54
Atrial fibrillation	5 (5.7%)	3 (3.4%)	.72
History of PCI	15 (17.2%)	14 (16.1%)	>.99
History of CABG	5 (5.7%)	2 (2.3%)	.44
Access—radial	66 (75.9%)	77 (88.5%)	.16
Access—femoral^a^	21 (24.1%)	13 (14.9%)	.18
Bleeding	2 (2.3%)	3 (3.4%)	.68
Vasopressor use	3 (3.4%)	11 (12.6%)	.03
Mechanical support	2 (2.3%)	5 (5.7%)	.28
Electrical instability	11 (12.6%)	14 (16.1%)	.49
Intubation	3 (3.4%)	6 (6.9%)	.33

Data are n (%).

CABG, coronary artery bypass graft; ERPS, enhanced radiation protection system; PCI, percutaneous coronary intervention; STEMI, ST-elevation myocardial infarction.

a Multiple patients required both radial and femoral access for mechanical support devices and PCI, therefore access sites do not total to the number of study participants.

**Table 2:** Multivariable linear regression estimates for predictors of door-to-balloon time.

Variable	β coefficient (min)	95% CI	*P* value
Rampart^a^	1.36	−1.00 to 3.72	.26
Bleeding	2.15	−5.23 to 9.54	.57
Acute kidney injury	2.08	−3.20 to 7.37	.44
Intubation	4.19	−1.67 to 10.06	.16
Femoral access^b^	3.48	−0.07 to 7.03	.055

a Reference is lead apron.

b Reference is radial access.

Multivariable regression models were performed to determine whether different patient characteristics influenced D2B times. When adjusting for D2B times between the 2 systems by taking into account patient demographics, cardiac risk factors, access (femoral vs radial), and complications like bleeding and acute kidney injury, the ERPS was noninferior (Table 1, Central Illustration). Baseline clinical and procedural characteristics are shown in Table 1. Groups were generally well balanced, although vasopressor use was more common in the ERPS group (χ^2^ *P* = .03). These variables were included in the adjusted analyses. Using a simplified model (core covariates only), which adjusted for risk factors like age, hypertension, and diabetes, along with complications (bleeding and intubation) and access, again confirmed noninferiority of the ERPS with adjusted difference in D2B time (ERPS vs lead apron) of 1.51 minutes (95% CI, −0.86 to 3.97, *P* = .21).

**Central Illustration:**
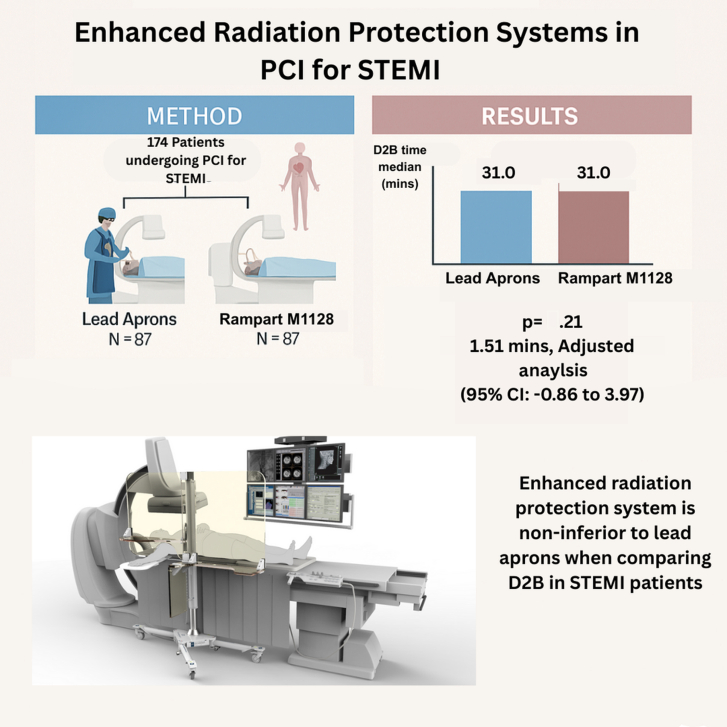
**Comparing Rampart M1128 with standard lead aprons for STEMI patients, highlighting no significant difference in door-to-balloon time when using the Rampart System.** D2B, door-to-balloon; PCI, percutaneous coronary intervention; STEMI, ST-elevation myocardial infarction.

Univariate covariate analysis (one-at-a-time) was performed to evaluate the influences of individual variables. Individual variables that could influence D2B time included bleeding, need for intubation, and access type (femoral vs radial) were analyzed. Even when analyzing factors one-at-a-time against the group variable, all CIs fell within the noninferiority margin of 5 minutes. Bleeding resulted in a group effect adjustment of 1.11 minutes (95% CI, −1.28 to 3.50; *P* = .36). Need for intubation group effect is a change of 1.29 minutes (95% CI, −1.10 to 3.68; *P* = .29). Access (femoral or radial) resulted in a group effect of 1.21 minutes (95% CI, −1.18 to 3.60; *P* = .32).

To analyze a potential learning curve with the system, linear regression was performed using procedure date and D2B times. This yielded a nonsignificant downward trend (β = −0.01 minutes per day, *P* = .27). This corresponded to an estimated reduction of 0.66 minutes over a 2-month period. Given the weak effect size and poor model fit (R^2^ = 0.014), there was no compelling evidence of a learning curve or improved D2B times once staff increased familiarity with the system, impacting the primary outcome.

All analyses resulted in the same conclusion, regardless of whether patient-level covariates were included. Use of an ERPS is noninferior to the use of traditional lead aprons when comparing D2B times during STEMI care.

## Discussion

This study sought to evaluate the effect of implementing an ERPS, in our case, Rampart M1128, on D2B time in STEMI patients. To our knowledge, this is the first study assessing the impact of such a system on D2B time. The key findings from the study are as follows: (1) there is no evidence that utilization of the Rampart M1128 system delays patient care in terms of D2B time, (2) this holds true despite the potential learning curve in the initial cases, and (3) patient and procedural factors including access type and presentation in cardiogenic shock or respiratory failure did not lead to a significant difference.

For decades, interventional cardiologists have worked tirelessly and diligently to improve the quality of care in STEMI patients by implementing systems to reduce D2B time. These include field STEMI activation, direct patient transport to the cath lab, app-based communication between emergency medical services (EMS) and cath lab teams, and establishment of regional STEMI networks, to name a few.^6^, ^7^, ^8^ These efforts are vital to improving outcomes, as the literature shows that time is muscle, with every 30-minute reduction in D2B time having a significant prognostic implication.^4^^,^^5^ On the other hand, there has been minimal effort in reducing the occupational hazards for interventional cardiologists until the introduction of this technology. A recently published survey of nearly 300 physicians practicing in cardiac cath labs across the world revealed a high prevalence of orthopedic injuries and cancer diagnoses.^10^ Several studies have been performed proving the effectiveness of radiation protection systems in reducing operator radiation,^11^, ^12^, ^13^ and although there are limited studies proving reduction of orthopedic strain, these are likely to emerge as more data become available.

The market of novel radiation protection systems is still in its infancy, with Rampart M1128 being one of the few commercially available systems in the United States. Initial systems, such as Zero-Gravity (Biotronik), focused on protecting the primary operator; however, more recent systems focus on protecting the entire catheterization laboratory staff. Protego (Image Diagnostics, Inc), EggNest XR (Egg Medical), and Rampart M1128 are the most prominent and have published data regarding their effectiveness at significantly reducing radiation exposure to the cath lab team, although mostly in studies with small sample sizes due to the novelty of the systems.^11^, ^12^, ^13^, ^14^ Radiation dosimeter readings were not collected in this analysis, and therefore, we cannot provide direct exposure comparisons between groups. However, multiple prior studies have shown that these systems significantly reduce operator radiation exposure.^11^, ^12^, ^13^, ^14^

To date, to our knowledge, there are no studies evaluating the impact of implementing such systems on D2B times in STEMI patients. As these systems become more prevalent, we must ensure that we are not compromising the quality of patient care. As with all new technologies, there is an expected learning curve. With Rampart, this involves draping the shields as well as positioning them over the patient and occasionally readjusting the position, depending on the fluoroscopic angles needed for coronary angiography. The patient’s body habitus, room size, access location, and presence of other devices also play a role in the speed of setup. Our center is a high-volume, tertiary care facility that receives transfers from many outlying hospitals, with most of our STEMI volume being transfers. To control for variability related to transfer time, our calculation started from the time of arrival at our facility to exclude transport-related delays, allowing for a more accurate measurement of D2B time. Our data suggest that there was no impact of a learning curve and that use of Rampart did not lead to significant delays in D2B time across all groups of patients, including those presenting in cardiogenic shock.

## Limitations

Given the novelty of the technology, our sample size is limited and only powered to test for a 5-minute difference in D2B time; however, the data suggest that the difference will be minimal, especially as the technology improves. Although the retrospective observational design carries inherent limitations, we mitigated these by including all-comer patients to reduce selection bias and multivariable regression analyses to adjust for key confounders. Despite inclusion of all-comers, the percentage of patients presenting in cardiogenic shock or requiring mechanical circulatory support/intubation was very low, which limits subgroup conclusions. This population would likely be the group with the largest delay related to the use of such systems due to the logistics of positioning the shields and obtaining large-bore access, although our data did not suggest that. Further studies with larger sample sizes would be needed to conclude this. Our high procedural volume allows for the quick development of proficiency in utilizing the system, which will translate to reduced time during STEMI presentations; therefore, our results may not be applicable to centers with lower volumes, although based on our limited data, the learning curve carries a <1-minute delay, which is quickly overcome.

## Conclusion

The results of our study reveal that implementation of an enhanced radiation protection system, in our case, Rampart M1128, does not significantly prolong D2B time in our single-center retrospective study of all-comers STEMI patients. Given the lack of negative impact on D2B time, we believe that health care systems should invest in protecting their cath lab staff, given the proven benefits without compromising the quality of patient care.

## Declaration of competing interest

The authors declared no potential conflicts of interest with respect to the research, authorship, and/or publication of this article.
